# Public Bodies and Workmen's Dwellings

**Published:** 1898-07-30

**Authors:** 


### PUBLIC BODIES AND WORKMEN'S DWELLINGS.

Among the various questions concerning the health and well-being of the working classes none are more important than those that have to do with their housing. Nothing can be more shocking than the manner in which too many of them live, not, in the first instance at least, of their own free will, and probably never so until habit has debased their sensibilities, and led them to prefer some immediate although fleeting gratification to the more permanent, but too often unattainable pleasure, of a clean and well ordered home. And the root of the whole difficulty is rent. The root of the overcrowding, and of the consequent dirt, sickness, and immorality, can generally be traced back in the first instance to rent, although it must in fairness be admitted that when overcrowding has once taken the edge off those instinctive feelings of nicety which rule the conduct of those who have never experienced its degrading influence, lowering of the rent will not by itself lead to a reversion to a more roomy mode of life. We insist upon the importance of rent because, whether the problem is to be solved by municipal or by private efforts, rent is its dominant factor.

On every side, and almost every week, we find individuals, associations, or conferences asking for municipal bodies to undertake the erection of workmen's dwellings, and that not so much for reasons of health as for reasons of rent. It is asserted that in some quarters there is an absolute dearth of such dwellings, even at the present rents, and it is openly urged that because public bodies can get money cheaply they should build dwellings so that they can be let to the working classes at lower rents than are now current. Nay, more; so that the rent may be still further brought down below what is possible to private enterprise, it is now asked that the term of years during which money borrowed for building purposes may be repaid may be extended. In every way it is being urged that the money powers of the municipal authorities should be used not merely for the sake of raising the standard of accommodation, but with the object of lowering the rent. The inevitable result of this must be to drive private enterprise out of the field, and to throw the entire task of providing dwellings for the working classes upon the municipal authorities. Are they prepared to take up this enormous task?

Putting altogether on one side for the moment all question of the justice of using for the benefit of one class rates contributed by all, this much at least is clear, that if dwellings are let below their market value not only is a present of so much per annum made to the fortunate occupiers, but a distinct wrong is done to all others of the same class who are excluded from participation in this bounty. It is easy to say that this will not happen, because the privately-owned houses will be forced by the competition of the municipal ones to come down in rent. But this result is not what would happen or, indeed, what is happening. If the population were stationary, and if a house, once a dwelling house, were bound to remain so, this might be; but such is not the case. There is a constantly increasing population to provide for, and there is a constant tendency for house owners to use their property for other purposes if their profits from letting them as dwellings are materially lessened; and, indeed, there is much reason to believe that a good deal of the present lack of dwellings for the working-classes is the direct outcome of the fact that capital is scared away from such investments because of the threatened wrecking of that kind of property by municipal competition. If the business of providing good sanitary houses for the working-classes were relieved from the threat of competition on such unequal terms, and if investors in such undertakings were assured, as they ought to be, that the powers of the sanitary authorities would be steadily exercised to ensure the closing of all insanitary property—the only property that could compete seriously with them,—the security offered by such investments would be so good that we may be perfectly certain that dwellings would be put up in such numbers as to be let at the lowest possible rental—not, perhaps, at so low a rental as can be afforded by a County Council, putting up a "model" dwelling here and there to show what can be done, but quite as low as the Council could afford if it were to take the whole business, with all its risks, upon its shoulders. The true function of a governing body is to govern, and there is every reason to believe that if the authorities would restrict their action to the enforcement of their sanitary bye-laws private enterprise would do all the rest. The County Council has far higher functions to perform than the providing of comfortable dwellings for the fortunate few who may happen to become its tenants. One cannot but feel that sanitary authorities will have a freer hand in dealing with insanitary dwellings if they themselves are not in the trade. It is already an evil that the County Council has been forced to crystallise in bricks and mortar a sanitary standard which it can hardly be doubted will in a few years be effete, and it is clear that every time this happens the hands of the Council's officers are to that extent tied in dealing with private owners and speculative builders.

